# An Unusual Cause of Persistent Wheeze in Infancy: Intrathoracic Neuroblastoma of the Lower Thoracic Sympathetic Chain

**DOI:** 10.1155/crpu/7447342

**Published:** 2026-06-03

**Authors:** Sara G. Hamad, Reem Bachir, Hiba Kammouh, Hoor Khedr, Juman A. Rezqalla, Mohammed Atea Al-Mashhadani

**Affiliations:** ^1^ Pediatric Pulmonology Department, Hamad Medical Corporation, Doha, Qatar, hamad.qa; ^2^ Pediatric Department, Sidra Medicine, Doha, Qatar, sidra.org; ^3^ Pediatric Department, Hamad Medical Corporation, Alwakra, Qatar, hamad.qa; ^4^ Pediatric Radiology Department, Hamad Medical Corporation, Alwakra, Qatar, hamad.qa

## Abstract

Neuroblastoma is the most common extracranial solid malignancy of childhood, typically arising from the adrenal medulla or paraspinal sympathetic chain. Although intrathoracic neuroblastoma is uncommon, involvement of the lower thoracic sympathetic chain is particularly rare, with most reported thoracic tumors arising in the upper posterior mediastinum rather than the lower paraspinal region. We report a 10‐month‐old boy diagnosed with intrathoracic neuroblastoma originating from the paraspinal region at the T12 level, which posed diagnostic challenges due to its rarity and nonspecific presentation. This case highlights the importance of including neuroblastoma in the differential diagnosis of intrathoracic masses in infants.

## 1. Introduction

Neuroblastoma is the most common extracranial solid tumor in children, accounting for approximately 7%–10% of all pediatric cancers and around 15% of cancer‐related deaths in this population [1]. It most commonly originates from the adrenal glands, although it can also arise from the sympathetic nervous system in other locations, such as the neck, chest, and pelvis [2]. The clinical manifestations of neuroblastoma are highly variable and often depend on the tumor’s location, size, and presence of metastasis. Common signs include abdominal mass, pain, and systemic symptoms like fever or weight loss. However, isolated respiratory symptoms, such as recurrent wheezing or persistent pulmonary opacity, are rarely seen in the absence of metastasis [3].

Adrenal neuroblastomas rarely extend superiorly to compress thoracic structures. When they do, it is usually associated with significant mass effect on adjacent organs, leading to respiratory complications like diaphragmatic elevation and atelectasis [4].

We here report a case of purely intrathoracic neuroblastoma arising from the lower thoracic sympathetic chain in an infant. This case report highlights the clinical challenge of diagnosing neuroblastoma in an infant presenting primarily with respiratory symptoms. The recurrent wheeze and persistent radiological opacity observed in this case were eventually linked to an intrathoracic neuroblastoma that exerted a mass effect on the lower thoracic structures.

## 2. Case Presentation

The patient is a 10‐month‐old male infant who was born at term to first‐degree consanguineous parents. He had a normal antenatal and perinatal course. At birth, he was admitted to the neonatal intensive care unit (NICU) due to neonatal respiratory distress and required continuous positive airway pressure (CPAP) support for 8 h. He was then weaned gradually to nasal cannula and discharged home on room air and oral feeding at 3 days of life as a case of transient tachypnea of the newborn. His initial chest X‐ray (CXR) showed non‐specific reticulogranular changes with no identified mass.

He was evaluated for recurrent admissions with lower respiratory tract infections (LRTIs). He had a total of three admissions, the first at the age of 3 months. At this presentation, he had cough and increased work of breathing, and was found to have hypoxia and wheezing. He was admitted to the pediatric intensive care unit (PICU) as a case of bronchiolitis with multiple viruses (adenovirus, influenza B, and rhinovirus) and required maximal respiratory support with high‐flow nasal cannula. The CXR revealed right upper and left lower zone haziness. Post‐discharge, he recovered completely with no cough, wheezing, or tachypnea.

**Figure 1:**
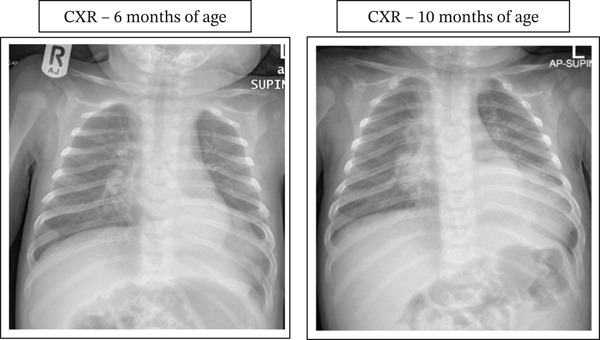
Anteroposterior chest X‐ray showing persistent left lower lobe opacity.

The second admission occurred at the age of 8 months. He was admitted with rhinovirus LRTI and virus‐induced wheeze exacerbation. He was tachypneic, wheezing, and hypoxic. CXR showed a left paracardiac opacity (Figure 1A). The patient was managed with oxygen support, bronchodilators, systemic steroids, and oral antibiotics with only partial response. He was discharged on room air.

However, post‐discharge, the mother reported intermittent cough, wheezing, and tachypnea over the following 2 months. The cough was mainly dry with poor response to bronchodilators. He also had choking with thin liquids.

He was hospitalized for the third admission at the age of 10 months. His respiratory viral PCR was negative. The child had no history of recurrent vomiting, diarrhea, recurrent ear infections, oral thrush, or weight loss, and no history of witnessed choking on a foreign body. Family history was notable for a brother with recurrent LRTIs and chronic aspiration syndrome.

On examination at the third presentation, the patient had a wet‐sounding cough but was not in distress. Audible wheezes were appreciated bilaterally with decreased air entry at the left posterior lower zone. Otherwise, his examination was unremarkable, and no abdominal mass was appreciated. Blood investigations were reassuring. CXR (Figure 1B) showed persistent left lower zone paracardiac opacity.

**Figure 2:**
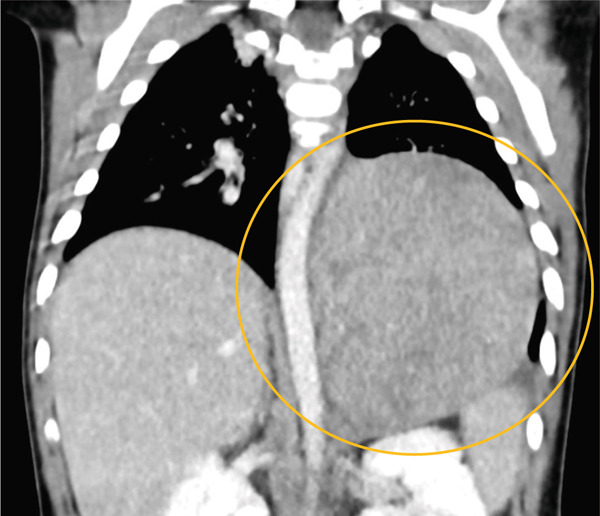
Coronal CT image revealing a large heterogeneously enhancing left intrathoracic/upper abdomen mass (yellow circle) with calcifications and superior extension compressing the left hemidiaphragm and adjacent lung parenchyma, resulting in lower lobe atelectasis.

Given the persistence of clinical and radiological findings, contrast‐enhanced computed tomography (CT) of the chest and abdomen (Figure 2) was performed to further evaluate the opacity. CT revealed a large left upper abdominal mass extending from the kidney to the hemidiaphragm. The lesion displayed internal coarse calcifications and heterogeneous post‐contrast enhancement. The left adrenal gland was not separately visualized, suggesting an adrenal origin. Findings were suggestive of neuroblastoma. Left lower lobe atelectasis was also noted due to elevation of the left hemidiaphragm.

**Figure 3:**
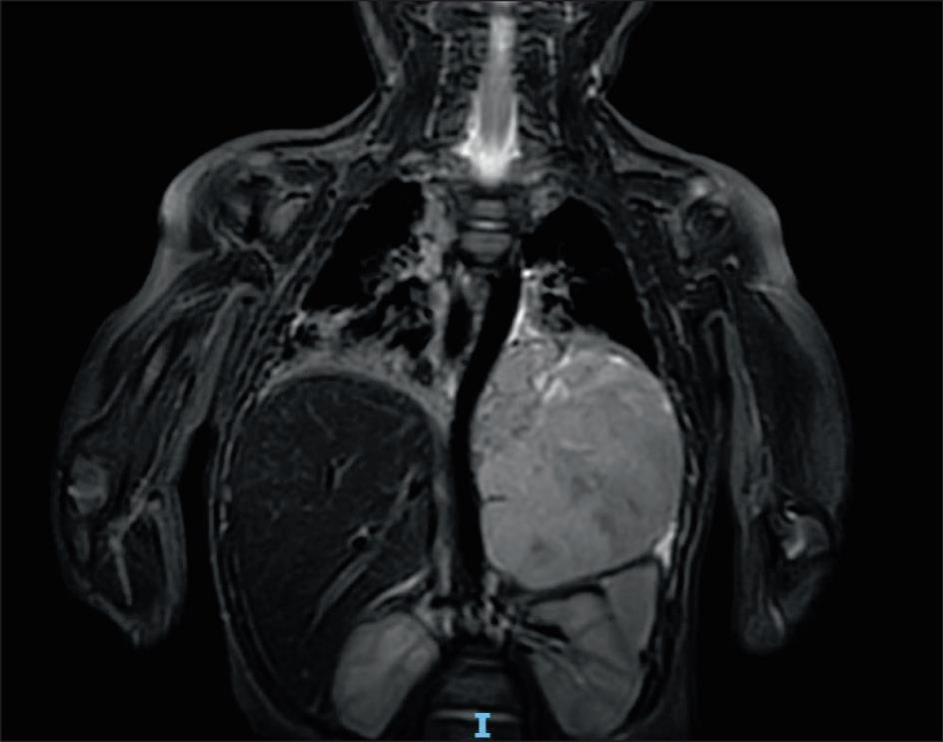
Magnetic resonance imaging (MRI) revealing large heterogeneous fairly‐well encapsulated lobulated enhancing left intrathoracic vs. suprarenal solid mass measuring approximately 6.0 × 6.9 × 8.2 cm.

Further investigations revealed elevated urinary catecholamines (VMA/Crea ratio: 117.2 mmol/mol [0.0–10.7] and HVA/Crea ratio: 205.3 mmol/mol [0.0–20.2]). Magnetic resonance imaging (MRI) showed a large heterogeneous, fairly well‐encapsulated, lobulated, enhancing left suprarenal/retroperitoneal solid mass measuring approximately 6.0 × 6.9 × 8.2 cm, initially suspected to be suprarenal on imaging; however, operative findings confirmed a purely intrathoracic paravertebral origin. It was hyperintense on T2WI, isointense to slightly isointense on T1WI, with small scattered areas of high signal likely representing blood products. It demonstrated extensive diffusion restriction with small non‐restricting areas likely representing minimal intertumoral necrosis. No definite intraspinal extension was seen on body imaging (Figure 3).

The findings were compatible with a primary adrenal neuroblastoma without further extension or metastases. Staging and resection were performed abroad at another medical facility. Core biopsy from the mass confirmed neuroblastoma, Schwannian stroma–poorly differentiated histology.

Molecular analysis revealed near‐pentaploidy (5n) with multiple chromosomal gains and losses detected by SNP array analysis. No MYCN amplification or segmental aberrations of 1p/q, 11q, or 17q were detected by FISH analysis.

No evidence of metastases was detected on bone marrow examination or positron emission tomography/computed tomography (PET/CT). Metaiodobenzylguanidine (MIBG) scintigraphy revealed low‐grade peripheral uptake.

The patient was diagnosed as having low/intermediate‐risk neuroblastoma amenable to surgical excision. Left thoracotomy and radical excision were performed. An intrathoracic paravertebral lesion was located in the left hemithorax, abutting the left diaphragm and measuring 8 × 7 × 6.3 cm, extending from the level of thoracic vertebra 5 (T5) to lumbar vertebra 1 (L1).

The patient recovered smoothly postoperatively. Upon follow‐up, he remained in remission. He had intermittent viral‐induced wheeze, well controlled with inhaled corticosteroids and bronchodilators, with infrequent exacerbations and complete interval resolution. Serial chest radiographs showed only minimal peribronchial changes.

## 3. Discussion

This case illustrates a rare and diagnostically challenging presentation of neuroblastoma in an infant. The predominant respiratory symptoms and persistent radiologic abnormalities led initially to a pulmonary‐focused diagnostic approach. The initial radiological images findings, including lobar atelectasis and persistent unilateral thoracic changes, were initially interpreted in the context of more common pediatric respiratory conditions such as chronic aspiration or post‐infectious sequelae. However, residual symptoms despite appropriate management prompted further investigations, ultimately revealing an intrathoracic neuroblastoma causing airway compression and lobar collapse.

Neuroblastoma in infancy often presents with non‐specific clinical features. It can also demonstrate variable imaging appearances depending on their site of origin, size, and relationship to adjacent structures [3]. In our case, the respiratory‐dominant presentation underscores how thoracic neuroblastoma may masquerade as primary pulmonary pathology, particularly when the tumor arises in an atypical paraspinal location.

Neuroblastomas in children originate from primordial neural crest cells committed to sympathetic differentiation and may therefore arise anywhere along the sympathetic chain [5]. The adrenal medulla is the most common site of origin, followed by the extra‐adrenal retroperitoneum. Thoracic neuroblastomas account for a smaller proportion of cases and typically arise from the posterior mediastinum along the upper thoracic sympathetic chain. Tumors originating from the lower thoracic sympathetic chain are distinctly uncommon, and this atypical location may result in overlapping imaging features that obscure the primary site of origin, as occurred in our patient. The inability to clearly identify the ipsilateral adrenal gland on CT and MRI contributed to diagnostic uncertainty and illustrates a known imaging pitfall in neuroblastoma localization [5].

Accurate staging of neuroblastoma relies heavily on imaging and is currently based on the International Neuroblastoma Risk Group Staging System (INRGSS). INRGSS was developed to enable pre‐operative, imaging‐based staging, independent of surgical findings [6, 7]. INRGSS classifies tumors based on the presence or absence of image‐defined risk factors (IDRFs). Under this system, stage L1 tumors are localized lesions confined to a single body compartment without IDRFs, while stage L2 tumors are locoregional lesions associated with one or more IDRFs, such as encasement of major vessels, airway compression, or intraspinal extension. Stage M denotes distant metastatic disease, excluding regional lymph nodes, whereas stage MS describes metastatic disease confined to the skin, liver, and/or bone marrow in infants younger than 18 months.

Based on pre‐operative imaging in our case, the tumor was classified as INRGSS stage L2. Cross‐sectional imaging demonstrated significant mass effect on adjacent thoracic structures, including elevation of the left hemidiaphragm and secondary left lower lobe atelectasis, consistent with airway and pulmonary compression. In addition, the tumor extended across the thoracoabdominal junction from T5 to L1, increasing surgical complexity. No intraspinal extension, major vascular encasement, or distant metastatic disease was identified. These findings underscore the critical role of imaging in identifying IDRFs and guiding risk stratification and surgical planning in neuroblastoma.

Thoracic manifestations in neuroblastoma are not limited to primary thoracic tumors. Prior imaging‐based studies have demonstrated that abdominal neuroblastomas may be associated with thoracic sequelae such as pleural effusion, likely secondary to diaphragmatic irritation or lymphatic obstruction [7]. These findings reinforce the need for radiologists to maintain a broad differential diagnosis when evaluating persistent or unexplained thoracic abnormalities in infants, even in the absence of an obvious thoracic primary lesion.

In our case, chest radiography served as the initial imaging modality that identified persistent abnormalities and prompted escalation to advanced imaging. CT and MRI were essential for characterizing the lesion, assessing its paraspinal origin, and evaluating its relationship to adjacent thoracic and abdominal structures. This stepwise imaging approach underscores the critical role of radiologic vigilance and multimodality imaging in the early detection of atypical neuroblastoma presentations but can also be inconclusive.

Management of neuroblastoma is highly individualized and reflects the marked heterogeneity in tumor location, stage, and biological behavior. Therapeutic strategies range from active surveillance to multimodal treatment, including surgical resection, chemotherapy, radiotherapy, stem cell transplantation, and immunotherapy. Low‐risk neuroblastoma typically presents as localized disease, particularly in infants, and is notable for its potential for spontaneous regression. In selected infants with small, asymptomatic tumors, close observation with serial imaging at regular intervals may be appropriate, allowing avoidance of surgical intervention [4].

For larger localized tumors or those diagnosed beyond infancy, surgical resection remains the cornerstone of treatment. In infants younger than 18 months, an observational approach for selected localized tumors is currently under investigation by international cooperative groups, while symptomatic patients may receive limited chemotherapy without routine use of radiotherapy or surgical palliation [4].

Intermediate‐risk neuroblastoma is characterized by limited metastatic involvement, such as regional lymph nodes or bone marrow in infants, and is generally managed with chemotherapy, with surgical resection considered when feasible [8].

High‐risk neuroblastoma carries the poorest prognosis and typically presents with widespread metastatic disease involving the bone marrow, bone, liver, or lungs. Management requires intensive multimodal therapy, including induction chemotherapy, maximal surgical resection, myeloablative chemotherapy with autologous stem cell rescue, followed by maintenance therapy incorporating immunotherapy [9]. The anti‐GD2 monoclonal antibody dinutuximab has become a key component of treatment, demonstrating improved event‐free survival in high‐risk patients [10].

A 20‐year single‐center experience demonstrated that implementation of the INRG staging system was associated with a shift toward increased use of minimally invasive surgical techniques and more standardized timing of surgery, particularly for extra‐adrenal neuroblastoma. Importantly, while surgical approaches evolved, the overall role of surgery in neuroblastoma management remained unchanged, reflecting adaptation to imaging‐based risk stratification rather than reduced surgical involvement [11].

Prognosis in neuroblastoma is highly variable and depends on a combination of age at diagnosis, disease stage, imaging features, and tumor biology [3]. Infants younger than 18 months generally have significantly better outcomes, particularly in the absence of metastatic disease. Imaging‐based staging plays a pivotal role in prognostication, as localized or locoregional tumors without distant spread are associated with favorable survival. Tumor biology further refines risk assessment, with absence of MYCN amplification and lack of segmental chromosomal aberrations involving 1p, 11q, or 17q conferring a favorable prognosis [12]. Fortunately, in our patient, the young age, localized disease, favorable molecular profile, and successful complete resection collectively contributed to classification as low‐ to intermediate‐risk neuroblastoma. These favorable prognostic features contributed to the decision and successful complete surgical resection and an excellent early clinical outcome.

## 4. Limitations

The rarity of neuroblastoma arising from the lower thoracic sympathetic chain limits the availability of imaging‐based comparative data and constrains broader radiologic generalization. In addition, long‐term follow‐up imaging is not yet available, precluding assessment of delayed treatment‐related changes or late recurrence patterns. Nevertheless, this case provides valuable radiologic insight into an uncommon anatomical presentation and highlights important imaging considerations when evaluating infants with persistent or atypical thoracic findings.

## Funding

No funding was received for this manuscript.

## Ethics Statement

This study was approved by the Hamad Medical Corporation Medical Research Center (MRC Approval Number: MRC‐04‐26‐066). Written informed consent for participation and publication of the clinical details and accompanying images was obtained from the patient’s guardian.

## Conflicts of Interest

The authors declare no conflicts of interest.

## Data Availability

The data that support the findings of this study are available on request from the corresponding author. The data are not publicly available due to privacy or ethical restrictions.
